# Unique challenges for appropriate management of a 16-year-old girl with superior mesenteric artery syndrome as a result of anorexia nervosa: a case report

**DOI:** 10.1186/1752-1947-3-127

**Published:** 2009-11-16

**Authors:** Philip A Verhoef, Angelika Rampal

**Affiliations:** 1 Department of Internal Medicine, University of California, Los Angeles, Le Conte Ave, Los Angeles, CA, USA; 2 Department of Pediatrics, Mattel Children's Hospital at the University of California, Los Angeles, Le Conte Ave, Los Angeles, CA, USA

## Abstract

**Introduction:**

Nausea and vomiting in an adolescent, though common presenting symptoms, often pose a diagnostic and therapeutic challenge to the physician. When the diagnosis involves both medical and psychiatric components, management can be complex, especially in the current healthcare system in the United States. To the best of our knowledge, there have been no previous publications detailing successful management of a patient with anorexia nervosa and superior mesenteric artery syndrome.

**Case presentation:**

We report the case of a 16-year-old Caucasian girl who presented to our emergency department with nausea, abdominal pain, diminished appetite and vomiting. Her history and examination were notable for a 15 kg weight loss and diffuse abdominal tenderness. A barium swallow X-ray with small bowel follow-through and computed tomography scan demonstrated remarkable duodenal narrowing between the superior mesenteric artery and the aorta, consistent with superior mesenteric artery syndrome. Initial management focused on relieving the obstruction and supporting the nutritional needs of the patient. Further history confirmed a diagnosis of anorexia nervosa, requiring intensive psychiatric and medical management, and necessitating a multifaceted approach to patient care involving social work, multiple primary care physicians and subspecialists, insurance company representatives, and the patient's immediate family.

**Conclusion:**

This case illustrates important points regarding the pathogenesis of superior mesenteric artery syndrome in the setting of anorexia, and it highlights the complexities that arise when managing an adolescent with both medical and psychiatric needs, as well as outlining a viable solution. While superior mesenteric artery syndrome is an uncommon cause of small bowel obstruction, the general pediatrician and child psychiatrist should be aware of this complication of anorexia nervosa.

## Introduction

In this case report, we describe an adolescent girl who presented to the emergency department with the common symptoms of nausea, vomiting, and abdominal pain, but who was found to have a small bowel obstruction and anorexia nervosa. The small bowel obstruction occurred as a result of weight loss secondary to the anorexia, which then led to loss of the omental fat pad and consequent compression of the duodenum between the superior mesenteric artery (SMA) and the aorta. While this particular association has been described previously, appropriate management, especially in the setting of the current US healthcare system, has not. Specifically, the adolescent had two distinct diagnoses - each was potentially life-threatening, yet appropriate management for one diagnosis seemed mutually exclusive of appropriate management for the other. We therefore describe various treatment options for managing both anorexia and SMA syndrome, and illustrate how we utilized a variety of resources to develop a novel and appropriate care strategy for this patient.

SMA syndrome was first described in a case report in 1842, and has since been described under various other names, including Wilkie disease, duodenal arterial mesenteric compression, duodenal ileus, and megaduodenum [1-3]. SMA syndrome is generally felt to result from compression of the duodenum between the superior mesenteric artery anteriorly/dorsally and the aorta (and behind the aorta, the vertebral column) posteriorly/ventrally [4]. This compression results from the loss of fatty tissue, which surrounds the superior mesenteric artery and its neurovascular pedicle. This can be a result of either weight loss or a rapid growth spurt. In the absence of an appropriate fatty scaffolding, the angle at which the SMA branches from the aorta is reduced resulting in compression of the third portion of the duodenum between the SMA and the aorta [4].

In our patient, SMA syndrome resulted from weight loss associated with anorexia nervosa. The Diagnostic and Statistical Manual of Mental Disorders, 4th Edition (DSM IV) diagnosis of anorexia nervosa is based on a patient meeting four criteria [5]: (1) the patient refuses to maintain a normal body weight, thereby resulting in a body weight of less than 85% of the ideal body weight; (2) patients have an intense fear of gaining weight or becoming fat; (3) the patient has a sense of body image dysmorphism; and (4) presence of amenorrhea in women (the absence of at least three consecutive menstrual cycles). Diagnosis, treatment and complications have recently been reviewed, and given that the standardized mortality ratio of patients with anorexia nervosa compared with the general population is 11.6 for all causes of death and 56.9 for suicide, anorexia nervosa remains a very important disorder to recognize and treat [5].

Though the incidence of SMA syndrome is thought to be quite low, the risk to the patient of complete obstruction forces the clinician to have a high index of suspicion in those patients who present with bilious vomiting and weight loss. Patients may have a history of early satiety related to delayed gastric emptying. However, a complete differential, including malrotation with volvulus, obstruction secondary to adhesions or masses, pancreatitis, peptic ulcer disease and motility disorders must be considered. Useful imaging includes a plain film of the abdomen that may reveal a 'double bubble' indicating duodenal obstruction, an upper gastrointestinal (GI) study that shows failure of contrast to flow distal to the third portion of the duodenum, and a computed tomography (CT) or magnetic resonance imaging/arteriography (MRI/MRA) scan with contrast demonstrating duodenal compression between the SMA and aorta [4].

Management begins with immediate attention to the small bowel obstruction, namely decompression via nasogastric (NG) tube placement and surgical consultation. After stabilization, management of SMA syndrome ranges from conservative attempts to increase weight with enteral tube feedings or parenteral nutrition, to more aggressive surgical therapy. Given that the gut is fully functioning distal to the site of obstruction, enteral tube feedings are preferred to parenteral nutrition, which has multiple side effects, including cholestasis. Surgical therapy may involve duodenojejunoanastomosis or gastrojejunoanastomosis aimed at bypassing the area of obstruction. Alternative surgical management may be removal of the ligament of Treitz in order to relieve the obstruction and allow greater intestinal mobility. Surgical management is necessary if medical management does not relieve the obstruction, if a nasojejunal (NJ) tube cannot be passed distal to the compression, or if the compression is not relieved by repletion of fat stores [1,2].

Medical management usually consists of tube feeds. A NJ tube can be progressed across the site of obstruction to provide enteral feeds. Feeds should be started at a low rate and advanced slowly in order to prevent refeeding syndrome, seen in individuals who quickly transition from a state of malnourishment to feeding. Electrolytes should be monitored closely, especially for hypophosphatemia, which can cause cardiac failure [2].

Weight gain must initially be followed daily, and then monitored at regular intervals by the pediatrician and specialists involved. The goal is to replete fat stores to decompress the third portion of the duodenum and eventually transition to oral feeds. Once on a stable regimen, the tube feeds can be given at home. The patient can ultimately be challenged with small amounts of ice chips and then liquids once he/she has begun regaining weight. Oral food intake can be advanced while simultaneously treating the underlying cause for the patient's SMA syndrome. All management should take place in consultation with GI, surgery, and adolescent specialists [2].

## Case presentation

We report the case of a 16-year-old Caucasian girl who presented to the emergency department complaining of severe nausea, epigastric pain, decreased appetite and vomiting.

Two weeks before admission, she experienced an acute onset of nausea, non-bilious vomiting, and diarrhea, which resolved within 24 hours. During the week before admission, she had an intermittent, dull, diffuse abdominal pain and decreased appetite. Over the few days before presentation, she had non-bilious emesis that progressed to bilious emesis. Her pain was non-radiating, sharp in nature and localized to the epigastric area. The pain was relieved with vomiting but it worsened after attempting to take in solids or liquids. She denied fevers, upper respiratory infection symptoms, hematemesis, hematochezia, or melena.

Further questioning revealed that during the 16 months before presentation, she had lost 22 kg (49 pounds). Upon questioning of both the patient and her mother, it was discovered that she had deliberately eliminated fat and meat from her diet and increased the amount of exercise she engaged in, including playing soccer and running. She described her body as 'sub-par' and she felt that she was challenging herself to be 'better'. She called herself a 'perfectionist' and was easily stressed out by school. However, she denied having a fixation with body image, although her mother privately reported that she had shown preoccupation with dieting, nutrition, and exercise. With her new diet and exercise regimen, she began to lose weight and she stated that she enjoyed the compliments she received regarding her physical appearance. She reported taking in approximately 900 calories per day and exercising more vigorously: she was running 3.5 miles three times a week and engaged in yoga for 1.5 hours a day on non-running days and was also playing soccer. As she continued on her diet and exercise regimen, she eventually reached a weight of 40 kg, down from her baseline of 61 kg. At that time, she was confronted by family and friends regarding her extreme thinness. She worked with her mother and her pediatrician in an attempt to increase her caloric intake and reduce her level of exercise. She acknowledged, on hospital admission, that she never ate more than 1800 calories a day, despite the agreements she made with her pediatrician and mother. Of note, she stopped having menses over a year before presentation.

Her physical examination on admission was notable for bradycardia (pulse ranging from 45 to 55), with a weight of 39 kg (z-score for age: -2.5), height of 161 cm (z-score -0.5) and body mass index (BMI) of 15 (z-score -2.9). The girl was cachectic and did not look well. Her head and neck examination was normal, including normal dentition. Her lungs were clear and she had normal respiratory effort. Her abdominal examination revealed diffuse tenderness to palpation and hypoactive bowel sounds. There were no masses or organomegaly. The rest of her examination was unremarkable.

**Figure 1:**
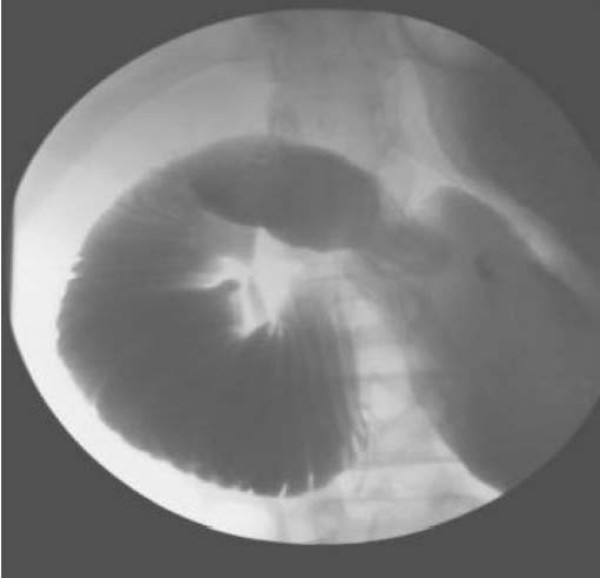
**Barium swallow X-ray with small bowel follow-through demonstrating obstructed flow of contrast at the mid-third portion of the duodenum**.

**Figure 2:**
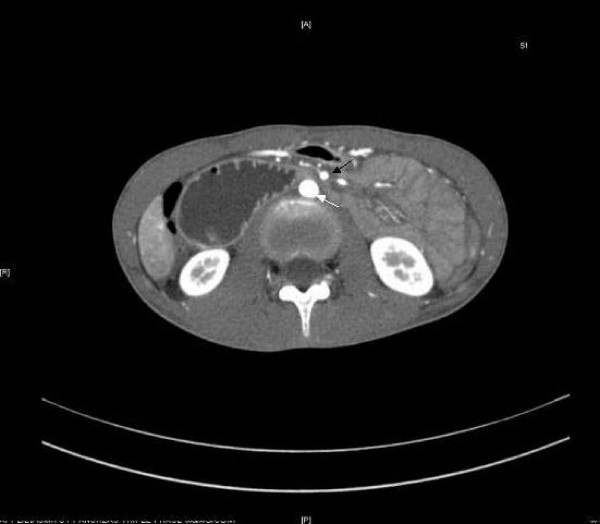
**Computed tomography scan demonstrating compression of the duodenum between the patient's aorta (white arrow) and superior mesenteric artery (black arrow)**.

Her evaluation in the emergency department included a barium swallow X-ray with small bowel follow-through that demonstrated an extremely dilated stomach and an inability of contrast to flow past the third portion of the duodenum (Figure 1). A CT scan also revealed a dilated stomach and proximal duodenum in addition to severe narrowing of the third portion of the duodenum. Axial contrast-enhanced CT during the arterial phase clearly demonstrated compression of the duodenum between the superior mesenteric artery and the aorta (Figure 2). The patient underwent immediate nasogastric tube decompression, resulting in relief of her epigastric pain and drainage of approximately 100 ml of bilious fluid. Laboratory evaluation at admission included electrolytes that were significant for a reduced chloride (95 mmol/l) and an elevated bicarbonate (36 mmol/l), consistent with emesis and a subsequent contraction alkalosis. Her amylase was 141 U/l, and lipase was 164 U/l, and her complete blood count and liver function studies were essentially normal.

After immediate decompression of her small bowel obstruction and consultation with pediatric surgery, an NJ tube was placed to provide nutrition beyond the site of obstruction. The pediatric surgical service felt that surgery would not be necessary if medical management could replete the patient's neurovascular fat stores to relieve the duodenal compression and prevent future compression. She was started on a tube feed regimen that provided 1800 kcal/day (47 kcal/kg/day), and was gradually titrated up to provide maximal caloric support, while monitoring for refeeding syndrome. Her ideal weight, based on a BMI of 20, was 54 kg, and we felt that she should regain at least 3 kg before initiation of oral feeds. The child psychiatry service diagnosed anorexia nervosa, restricting type, based on her weight restriction for 16 months, her excessive exercise, her low BMI, and her preoccupation with nutrition and exercise. They recommended transfer to an in-patient eating disorders unit, but her requirement for NJ feeds precluded this transfer. They felt that she should remain hospitalized until the NJ tube could be removed, followed by transfer to the eating disorders unit, but the patient and her family were not comfortable with this plan. Ultimately, after many multidisciplinary meetings involving nursing, discharge planners, insurance company representatives, the family, and the involved physicians, an out-patient eating disorder therapist was identified who agreed to provide regular counseling three times per week. This allowed the patient to be discharged home on tube feeds, while still receiving appropriate psychotherapy for her anorexia.

Six weeks after hospital discharge, the patient was tolerating approximately 1600 kcal/day of NJ-tube feeds and 1300 kcal/day of a diet consisting of protein shakes, yoghurt, and soft foods, without nausea, vomiting, or constipation. She transitioned to a 3000 kcal/day oral diet without difficulty and the NJ tube was discontinued shortly thereafter. Once the NJ tube was removed, she enrolled in an out-patient eating disorders day program, where she continued to receive intensive daily therapy for more than a month. She began having regular menstrual cycles again, and was able to maintain her weight at an appropriate level. One year after discharge from the hospital, her weight was 53 kg, and she remains an avid athlete and excellent student.

## Discussion

SMA syndrome in the setting of anorexia nervosa has been recognized, but there have been no published reports indicating how SMA syndrome might be successfully managed in the setting of anorexia [6]. There are two case reports which describe individual patients with anorexia nervosa and SMA syndrome who were unwilling to comply with psychotherapy and consequently underwent multiple endoscopies and gastrojejunostomy without improvement in their respective conditions [7,8]. Clearly, this combination of pathologies is uniquely challenging: SMA syndrome can precipitate and exacerbate anorexia nervosa because of the nausea associated with a small bowel obstruction and conversely, anorexia nervosa prevents the patient from being willing or able to ingest adequate calories to allow the SMA syndrome to resolve.

In our case, the patient no longer needed to be hospitalized for medical treatment alone as tube feeds could be given at home. Convincing the family and involved parties, however, that hospitalization was required in order to provide regular psychiatric follow-up for the potentially fatal disease of anorexia, was difficult. Various options were discussed: (1) that she remain hospitalized on the pediatrics ward for tube feeds, with monitoring and encouragement of caloric intake and weight gain, and treatment by the psychiatry consult service; (2) that she be transferred to the in-patient eating disorders unit while continuing NJ-tube feeds; (3) that she be discharged home, but monitored closely by her pediatrician, and that she be admitted to a partial psychiatric program with therapy from approximately 8.00 a.m. to 2.00 p.m.; (4) that she have the NJ tube removed in an attempt to begin liquid feeding and that she be immediately transferred to the in-patient eating disorders program; (5) that she have surgical correction, after which she could immediately resume oral feeds, and in-patient psychiatric evaluation; and (6) that she be discharged home with close follow-up by her pediatrician and an out-patient therapist, and that she be subsequently admitted to either the full in-patient or the partial in-patient eating disorders program after her weight had stabilized and the NJ tube could be removed.

We did not feel she should be subjected to the risks of surgical intervention solely to expedite her admission to the psychiatric unit if other non-surgical options existed. We were also concerned about the psychological and financial consequences to the patient and her family of prolonged hospitalization in an acute-care facility. The psychiatry service felt that they were ill-equipped to care for a patient receiving NJ-tube feeds on either an in-patient or out-patient service, and that the patient's NJ tube could have an adverse impact on other participants in group therapy sessions. Ultimately, an interdisciplinary effort was required to create a plan that included appropriate, high-level out-patient therapy for severe anorexia along with continued medical management by both the patient's pediatrician and gastroenterologist.

An ability to navigate the healthcare system via interdisciplinary management, including multiple subspecialists, nursing, discharge planning and insurance companies is now part and parcel of patient care. In this case, the members of the interdisciplinary team benefited from an actively involved discharge planner and insurance representative. In those patients who have minimal or no health insurance, psychiatric management becomes an even more difficult challenge given that many in-patient psychiatric units do not accept Medicaid insurances. As physicians are trained to advocate for patients and provide them with excellent care, it is fundamental to realize that the effectiveness of patient advocacy and patient care is intensified by the ability of the physician to know, understand, utilize and navigate the resources that are available in the various healthcare systems where we serve our patients.

## Conclusion

Nausea and vomiting, while common presenting symptoms, may represent a life-threatening illness with both medical and psychiatric etiologies. The clinician must be adept at recognizing the evidence of small bowel obstruction as well as the presence of mental illness that contributes to both the development of medical disease and complicates its treatment. This case report explores all of these issues and outlines an approach to therapy, which is multi-faceted and best serves the patient.

## Abbreviations

CT: computed tomography; DSM IV: Diagnostic and Statistical Manual of Mental Disorders, 4th Edition; GI: gastrointestinal; MRI/MRA: magnetic resonance imaging/arteriography; NG: nasogastric tube; NJ: nasojejunal tube; SMA: superior mesenteric artery

## Consent

Written informed consent was obtained from the patient for publication of this case report and any accompanying images. A copy of the written consent is available for review by the Editor-in-Chief of this journal.

## Competing interests

The authors declare that they have no competing interests.

## Authors' contributions

PV worked under the supervision of AR in the care of this patient. PV and AR worked jointly together to develop the outline for this case report, review necessary data, generate the figures, and draft the final written version of the manuscript.
